# Lysosomotropism depends on glucose: a chloroquine resistance mechanism

**DOI:** 10.1038/cddis.2017.416

**Published:** 2017-08-24

**Authors:** Laura E Gallagher, Ohood A Radhi, Mahmud O Abdullah, Anthony G McCluskey, Marie Boyd, Edmond Y W Chan

**Affiliations:** 1 Strathclyde Institute for Pharmacy and Biomedical Sciences, University of Strathclyde, Glasgow, Scotland G4 0RE, UK

## Abstract

There has been long-standing interest in targeting pro-survival autophagy as a combinational cancer therapeutic strategy. Clinical trials are in progress testing chloroquine (CQ) or its derivatives in combination with chemo- or radiotherapy for solid and haematological cancers. Although CQ has shown efficacy in preclinical models, its mechanism of action remains equivocal. Here, we tested how effectively CQ sensitises metastatic breast cancer cells to further stress conditions such as ionising irradiation, doxorubicin, PI3K-Akt inhibition and serum withdrawal. Contrary to the conventional model, the cytotoxic effects of CQ were found to be autophagy-independent, as genetic targeting of ATG7 or the ULK1/2 complex could not sensitise cells, like CQ, to serum depletion. Interestingly, although CQ combined with serum starvation was robustly cytotoxic, further glucose starvation under these conditions led to a full rescue of cell viability. Inhibition of hexokinase using 2-deoxyglucose (2DG) similarly led to CQ resistance. As this form of cell death did not resemble classical caspase-dependent apoptosis, we hypothesised that CQ-mediated cytotoxicity was primarily via a lysosome-dependent mechanism. Indeed, CQ treatment led to marked lysosomal swelling and recruitment of Galectin3 to sites of membrane damage. Strikingly, glucose starvation or 2DG prevented CQ from inducing lysosomal damage and subsequent cell death. Importantly, we found that the related compound, amodiaquine, was more potent than CQ for cell killing and not susceptible to interference from glucose starvation. Taken together, our data indicate that CQ effectively targets the lysosome to sensitise towards cell death but is prone to a glucose-dependent resistance mechanism, thus providing rationale for the related compound amodiaquine (currently used in humans) as a better therapeutic option for cancer.

During macroautophagy (referred to herein as autophagy), cellular components are sequestered into double-bilayer membrane vesicles termed autophagosomes. Autophagosomes next undergo fusion with lysosomes to allow content degradation and recycling of metabolic building blocks to sustain cell viability.^1^ Autophagy generally helps promote cancer progression.^2, 3, 4^ Autophagy maintains a healthy pool of mitochondria, for example, in K-Ras dependent tumours^5, 6^ to support oxidative metabolism, fatty-acid oxidation and generation of anabolic precursors.^1, 7^ Autophagy also helps cancer cells endure chemo- and radiotherapy, thereby contributing towards resistance.^8, 9, 10^ As such, autophagy inhibitors are being investigated to enable better treatment of tumours.

Chloroquine (CQ) or its derivative hydroxychloroquine (HCQ) has been widely tested in preclinical cancer models as an inhibitor of the autophagy–lysosomal pathway. These antimalarial drugs have been attractive candidates for repurposing in cancer because of their low cost, oral availability and FDA approval. Initially, clear inhibitory effects from CQ were shown in a number of haematological cancers.^11, 12, 13^ Beneficial effects of CQ have been demonstrated for other solid cancer models.^8, 14, 15, 16, 17, 18, 19^ This body of evidence has supported over 70 clinical trials assessing safety and efficacy using CQ or HCQ (www.Clinicaltrials.gov).^2^ Other strategies have explored CQ derivatives.^20, 21, 22, 23^

Despite the substantial testing of CQ in cancer patients, its mechanism of action remains controversial. CQ was initially proposed as an autophagy inhibitor and this notion still persists.^24^ CQ acts as a weak base and accumulates in the lysosomes to quench the acidic pH,^25^ thereby halting autophagic degradative flux. However, CQ could be targeting cancer cells via autophagy-independent pathways.^19, 26, 27, 28^ Here, we studied CQ in an aggressive metastatic breast cancer model. CQ sensitised cells to a number of cell stressors and we found that CQ mediated cell killing independently of autophagy. In exploring metabolic stress, we discovered an unexpected mechanism of cellular resistance linking CQ sensitivity to glucose utilisation. We further identified that amodiaquine (AQ), a related anticancer quinoline, engages a mechanism distinct from CQ that is not inhibited by changes in glucose metabolism, thereby highlighting a potentially improved anticancer treatment strategy.

## Results

### CQ sensitises cells to a range of cellular stressors

**Figure 1:**
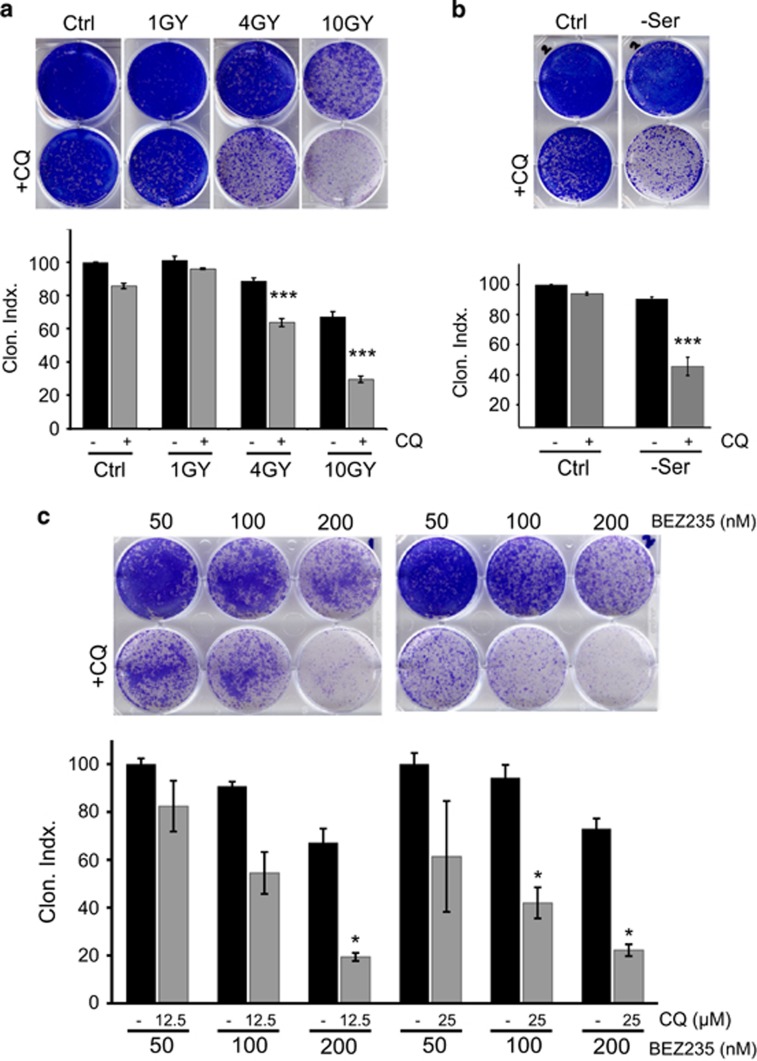
CQ sensitises 4T1 cells to ionising radiation, MTOR-PI3K inhibition and growth factor/serum depletion. (**a**) 4T1 cells were exposed to irradiation (1–10 Gray) and incubated ±CQ (25 *μ*M) as indicated for 24 h. After this treatment, drug-free media were replenished and viability was assessed by clonogenic growth and quantified. Viability expressed as clonogenic index where control (no CQ) represents 100% (*n*=3 experiments±S.E.M.). ***P<0.001 by one-way ANOVA as compared with no CQ at equivalent irradiation. (**b**) Cells were incubated in full-nutrient or serum-free DMEM ±CQ (25 *μ*M) for 24 h. Cells were then replenished with full-nutrient drug-free growth media and assessed for viability (*n*=3 experiments±S.E.M.). ***P<0.001 by one-way ANOVA as compared with no CQ serum-starved condition. (**c**) Cells were treated with the dual MTOR/PI3K inhibitor (NVP-BEZ-235) at increasing doses ±CQ for 24 h. Full-nutrient drug-free growth media were replenished and cells were assessed for viability (*n*=3 experiments±S.E.M.). *P<0.05 by one-way ANOVA as compared with paired no CQ serum condition under same NVP-BEZ-235 dose

CQ use in breast cancer has shown promise, but the full potential remained unclear.^9, 14, 26, 29^ As such, we explored combinations of CQ with range of anticancer treatments using 4T1 metastatic breast cancer cells. Incubation with CQ for 24 h only led to marginal (<15%) killing of 4T1 cells as detected by clonogenic survival (Figure 1a). Similarly, treatment of cells with 0–10 Gy X-irradiation alone induced only low-level, but dose-dependent, cytotoxicity in 4T1 cells (<40% killing after administration of the highest 10 Gy dose). However, incubation with CQ significantly sensitised cells to 4 or 10 Gy irradiation doses.

As CQ can sensitise to loss of PI3K/MTOR signalling,^30, 31, 32^ we further examined this combination in 4T1 cells. Inhibition of PI3K/MTOR, through serum starvation for 24 h, did not substantially reduce cell viability (Figure 1b). However, CQ enhanced levels of cytotoxicity produced from serum withdrawal. The dual PI3K/MTOR inhibitor NVP-BEZ235 acts as a robust anticancer compound, particularly for breast cancer.^33, 34, 35, 36, 37^ Here, BEZ235 alone was cytotoxic in 4T1 cells in a dose-dependent manner (Figure 1c). However, cytotoxicity for each dose of BEZ235 was potentiated by greater than threefold via co-addition of CQ. These findings show that CQ can strongly potentiate the cell death caused by DNA-damaging and growth factor deprivation stress conditions in breast cancer cells.

### CQ decreases viability via an autophagy- and apoptosis-independent mechanism

**Figure 2:**
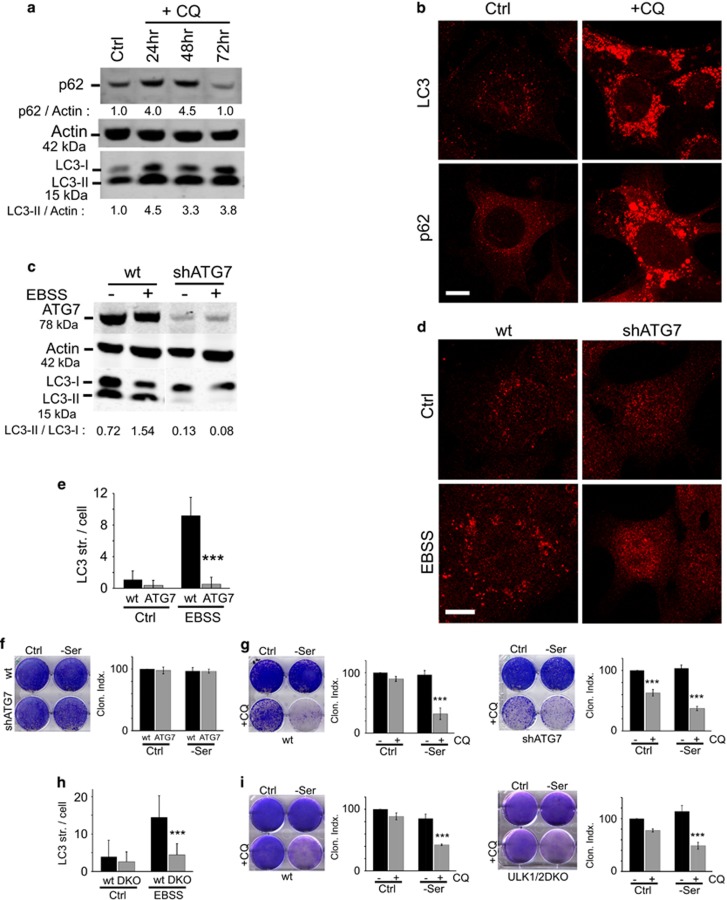
CQ sensitisation to cell death is independent of autophagy. (**a**) 4T1 cells were treated under full nutrient conditions with CQ (25 *μ*M) for up to 72 h. Cell lysates were analysed for LC3 and Sequestosome 1/p62 protein levels by immunoblotting. Quantification is expressed as fold change in LC3-II/actin or p62/actin. (**b**) 4T1 cells were treated as above with CQ for 24 h and stained for LC3- and p62-associated membranes. (**c–e**) 4T1 cells expressing ATG7 shRNA were treated to amino-acid starvation (EBSS incubation) for 2 h. Cell lysates were analysed for ATG7 levels and lipidation of LC3 (**c**) or autophagosome number by staining for LC3 (**d**; scale bars: 10 *μ*m). Autophagosomes in wild-type or shATG7-4T1 cells were quantified (expressed as mean±S.D. from >80 cells; two experiments). ***P<0.001 by paired *t*-test as compared with wild-type starved cells. (**f**) Wild-type or shATG7-expressing 4T1 cells were incubated in full-nutrient (Ctrl) or serum-free DMEM for 24 h before exchange back into fresh full-nutrient media to assess clonogenic viability. Genetic targeting of autophagy did not sensitise cells to serum starvation. All viability shown as Clonogenic Index relative to control (*n*=3 experiments ±S.E.M.). (**g**) Wild-type (left) or shATG7-(right) expressing 4T1 cells were incubated in full-nutrient (Ctrl) or serum-free DMEM ±CQ (25 *μ*M) for 24 h before exchange back into fresh full-nutrient media to assess clonogenic viability. ***P<0.001 by one-way ANOVA as compared with paired no CQ condition. CQ shows efficacy in autophagy-deficient 4T1 cells. (**h**) Wild-type or ULK1/2 DKO MEFs were analysed for LC3 autophagosome number following 1.5 h starvation in EBSS as in (**d** and **e**). Mean±S.D. from >90 cells; three experiments. ***P<0.001 by paired *t*-test as compared with wild-type starved cells. (**i**) Wild-type or ULK1/2 DKO MEFs were analysed as in (**g**). Genetic targeting of autophagy did not sensitise MEF, and ULK1/2-deficient cells are still targeted by CQ

CQ acts as a weak base to de-acidify the lysosome,^25^ thereby inhibiting autophagic degradative flux. However, the relationship between CQ-mediated cell viability and autophagy has become controversial.^18, 19, 26, 27, 28^ Here, we observed that CQ robustly inhibited the autophagy flux in 4T1 cells as observed through the accumulation of LC3 and p62/SQSTM1 protein and autophagy membrane structures staining positive for LC3 or p62/SQSTM1 (Figures 2a and b). Cells with autophagy inhibition were generally viable for up to 72 h under CQ treatment with sustained LC3 accumulation and no overt cell loss. Autophagy inhibition could, therefore, be tolerated without causing cytotoxicity.

To clarify whether CQ was decreasing cell viability through autophagy, we used shRNA to stably knockdown ATG7 in 4T1 cells.^2, 7^ 4T1 cells lacking ATG7 were fully defective for basal and starvation-induced LC3 lipidation (Figures 2c–e). Despite this complete block, 4T1/shATG7 cells were not more sensitive than wild-type 4T1 to serum starvation stress (Figure 2f). 4T1/shATG7 cells additionally retained sensitivity to CQ-induced cytotoxicity, both alone, or when combined with serum starvation (Figure 2g). As such, CQ led to cytotoxicity independently of autophagy. To investigate CQ-induced cytotoxicity in a different system, we used ULK1/2 double knockout (DKO) mouse embryonic fibroblasts (MEFs) that are defective in nutrient-sensitive autophagy (Figure 2h).^38^ We confirmed that wild-type MEF displayed sensitivity to CQ-induced cytotoxicity, in particular, under combination with serum starvation (Figure 2i). Loss of autophagy in ULK1/2 DKO MEF did not make them more sensitive to serum starvation. Furthermore, CQ combined with serum starvation still produced cell killing in ULK1/2-deficient cells. These data highlight that genetic targeting of autophagy does not sensitise cells to stress conditions in the same way as CQ. In addition, CQ-induced cell killing does not require autophagy.

**Figure 3:**
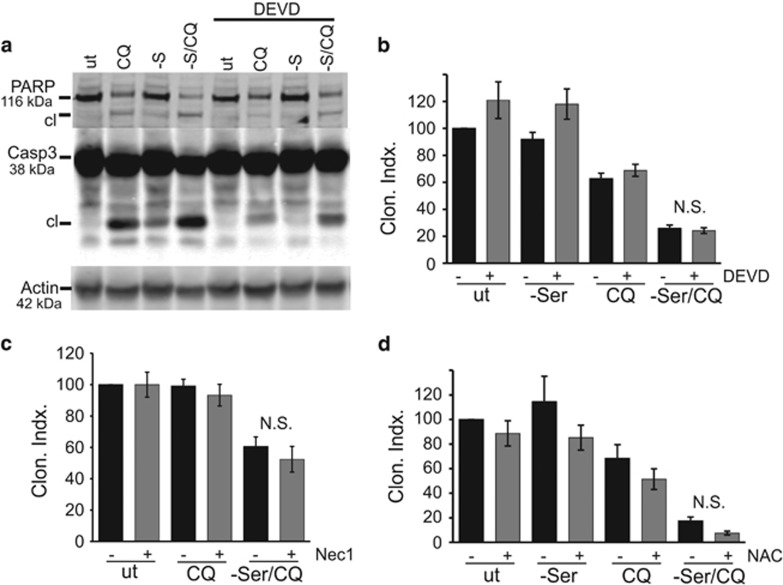
CQ triggers apoptosis- and necroptosis-independent 4T1 cell death. (**a**) Cells were starved of serum (-S) ±CQ (25 *μ*M) for 24 h. Where indicated, Z-DEVD-FMK (10 *μ*M) was included. Immunoblotting confirmed PARP and caspase 3 cleavage following CQ treatment combined with serum starvation. (**b**) Cells were starved of serum ±CQ (25 *μ*M) in the presence of Z-DEVD-FMK (10 *μ*M), as indicated, for 24 h. After treatments, clonogenic viability was measured. Z-DEVD-FMK did not significantly (N.S.) rescue cell viability despite blocking caspase 3 activation. (**c**) Cells were starved of serum ±CQ (25 *μ*M) in the presence of Necrostatin-1 (20 *μ*M), as indicated, for 24 h. The necroptosis inhibitor did not rescue cell viability. (**d**) Cells were starved of serum ±CQ (25 *μ*M) in the presence of *N*-acetyl-cysteine (10 mM), as indicated, for 24 h. ROS scavenger did not rescue cell viability

CQ and related compounds, particularly in combination with BEZ235, have been reported to activate apoptosis.^31, 39, 40, 41^ Either CQ or serum starvation alone in 4T1 cells led to low levels of caspase 3 activation and PARP cleavage (Figure 3a). Combination of CQ with serum starvation led to stronger caspase 3 activation, and all these apoptotic signalling responses were blocked by the inhibitor z-DEVD-FMK. However, z-DEVD-FMK could not reverse the cytotoxicity produced by CQ alone or when combined with serum starvation (Figure 3b).

As inhibition of caspase 3 did not ameliorate cell kill, we tested whether CQ invoked necroptosis, another mode of programmed cell death.^42, 43, 44, 45^ Necroptosis can be pharmacologically probed by necrostatin-1, which inhibits receptor-interacting kinase 1. Addition of necrostatin-1 was unable to rescue viability of 4T1 cells treated to CQ combined with serum starvation (Figure 3c). CQ and other lysosome-targeting agents have been associated with reactive-oxygen species (ROS) contribution towards cell death.^41, 44, 46, 47^ However, addition of free radical scavenger N-acetyl cysteine was unable to rescue cells treated to CQ combined with serum starvation (Figure 3d). Thus, CQ-mediated cytotoxicity was occurring independently of apoptosis, necroptosis, autophagy and ROS.

### Glucose starvation causes resistance to CQ-mediated cell killing

**Figure 4:**
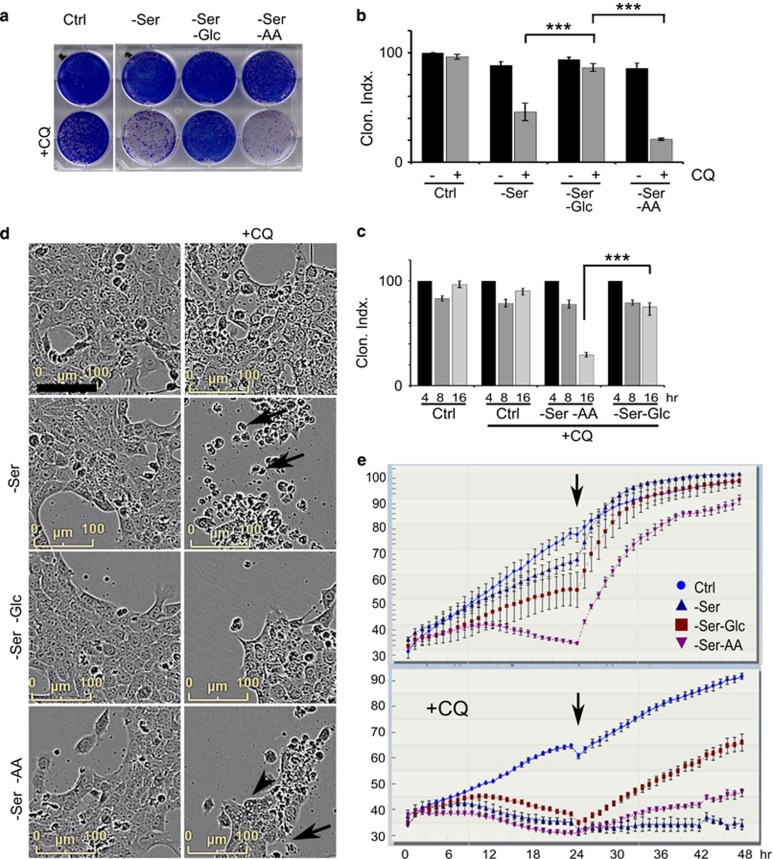
Glucose starvation rescues CQ-dependent cell death. (**a** and **b**) 4T1 cells were incubated in four different nutrient conditions: (1) full-nutrient DMEM (Ctrl); (2) serum-free DMEM; (3) serum- and glucose-free DMEM; or (4) serum- and amino-acid-free media (EBSS); ±CQ (25 *μ*M) as indicated. After 24 h incubation, clonogenic cell viability was measured (% of control, *n*=3 experiments±S.E.M.). ***P<0.001 by one-way ANOVA. (**c**) 4T1 cells were treated with the indicated nutrient conditions ±CQ for 4–16 h and then measured for cell viability as in (**a**) (*n*=3 experiments±S.E.M.). (**d** and **e**) 4T1 cell growth and morphology were monitored using live-cell imaging during nutrient and CQ treatments as indicated. (**d**) Images shown from the 23 h time point. Arrows indicate CQ-induced cell condensation and death. Arrowhead indicates vacuolated cell death. (**e**) Cell viability (% of imaging area containing cells, average ±S.E.M., *N*=2 experiments) are shown over the 48 h experiment. After 24 h nutrient ±CQ treatments, full-nutrient drug-free media was replenished (arrow) and cells were further monitored

As aggressive cancer cells (such as 4T1) become addicted to nutrients,^24^ we explored starvation and responses to CQ. We compared the following: (1) serum starvation; (2) serum and glucose starvation; and (3) serum and amino-acid starvation – all in the presence or absence of CQ. As control, we confirmed loss of cell viability when CQ is combined with serum withdrawal. Unexpectedly, further withdrawal of glucose, in the context of serum starvation, fully rescued the effect of CQ (Figures 4a and b). In contrast, withdrawal of amino acids and serum together promoted CQ-mediated cytotoxicity. CQ and nutrient starvation required more than 8 h to produce cytotoxicity. Incubation under the most stressful condition (serum and amino-acid starvation +CQ) produced >50% decreases in cell viability only after relatively long (16 h) incubation (Figure 4c). By comparison, glucose starvation and serum starvation (with CQ) did not lead to cell killing under the same timeframe. We confirmed that glucose starvation similarly protected wild-type and ULK1/2 DKO MEF from CQ-mediated cytotoxicity (Supplementary Figure 1).

Nutrient starvation combined with CQ seemed to be killing cells independently of classical apoptosis and necroptosis. To gain further insight into the underlying cell death, we monitored cell morphology by hourly live-cell imaging (Figure 4d and Supplementary Movies). Consistent with the viability time course, 4T1 cells incubated in serum starvation +CQ appeared morphologically normal for up to 16 h, after which cells exhibited rounding, condensation and detachment. Starvation of glucose along with serum blocked these CQ-induced changes. Quantitation of cell growth highlighted the varying responses to nutrients and CQ (Figure 4e). Serum starvation alone (top panel) was generally tolerated, with a mild decrease in cell growth over the 24-h starvation period. Cell growth was more strongly suppressed by combined glucose and serum starvation. Under combined amino-acid and serum starvation, reduced cell growth was displayed for 12 h, after which there was a further decline in cell proliferation, presumably after glucose had also been depleted. Interestingly, after all these 24-h starvation stress conditions, cells returned to a rapidly dividing state after replenishment of nutrients.

CQ treatment alone was non-toxic (bottom panel). By contrast, combination of CQ with any of the starvation conditions led to clear cell targeting within 12–18 h. As expected, cells stressed with CQ and serum starvation (or serum and amino-acid starvation) did not recover following replenishment of full nutrient drug-free media. However, cells that had glucose starvation showed resistance to CQ and recovered after nutrient replenishment and removal of CQ.

### 2-Deoxyglucose mimics glucose starvation in causing resistance

**Figure 5:**
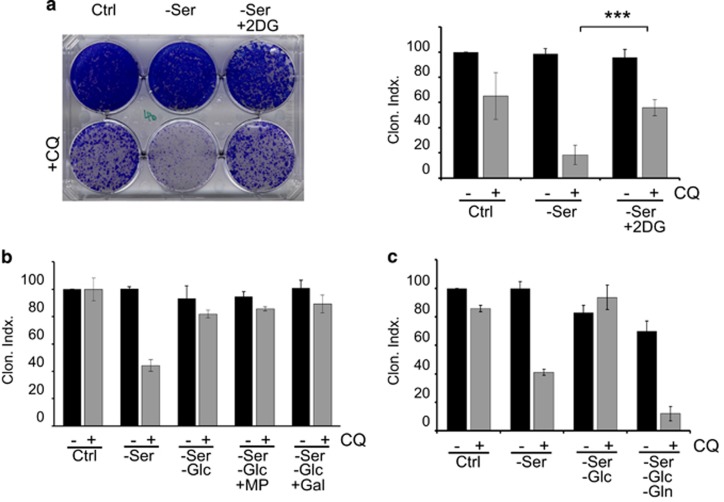
Inhibition of hexokinase rescues CQ-dependent cell death. (**a**) 4T1 cells were treated with full-nutrient DMEM or serum-free DMEM, in the presence of 2DG (5 mM) ±CQ (25 *μ*M) as indicated, for 24 h. After this incubation, cell clonogenic viability was measured (*n*=3 experiments ±S.E.M.). ***P<0.001 by one-way ANOVA. (**b**) Cells were treated with serum-free DMEM, or serum-free and glucose-free DMEM+CQ (25 *μ*M) for 24 h, followed by cell viability measurements. Where indicated, incubations included methyl pyruvate (10 mM) or galactose (Gal, 5 mM). (**c**) Cells were treated the following nutrient conditions: (1) full-nutrient DMEM; (2) serum-free DMEM; (3) serum- and glucose-free DMEM; or (4) serum-, glucose- and glutamine-free DMEM, ±CQ (25 *μ*M), as indicated for 24 h, followed by clonogenic viability measurements

In 4T1 cells, glucose starvation offered protection from CQ coupled with serum starvation. To further explore this relationship, we examined the glycolysis inhibitor, 2-deoxyglucose (2DG). 2DG becomes phosphorylated in cells to generate 2DG-6-phosphate, which inhibits the glycolytic enzymes hexokinase and phosphoglucose isomerase.^48^ Interestingly, addition of 2DG significantly promoted survival of cells under the stress of CQ in combination with serum starvation (Figure 5a). We tested whether other glycolysis inhibitors could confer the same protective advantage as 2DG. However, neither Gossypol (inhibitor of lactate dehydrogenase) nor dichloroacetate (inhibitor of pyruvate dehydrogenase kinase) was able to rescue 4T1 cell viability from CQ and serum starvation (Supplementary Figure 2A). 2DG is therefore the best pharmacologic agent so far tested that mimics glucose starvation in preventing the cytotoxicity of CQ.

Energy depletion activates an AMPK-dependent metabolic cell-cycle checkpoint that also promotes cell survival.^49, 50, 51, 52, 53^ Therefore, we tested whether increased cell survival upon glucose starvation was driven by AMPK. However, co-incubation with AICAR to activate AMPK did not rescue cell viability (Supplementary Figure 2B). We further questioned whether increased survival was due to overall suppressed levels of metabolism. We tested the cell-permeable intermediate, methyl pyruvate (MP), which is converted to acetyl-CoA to drive oxidative phosphorylation.^54^ As an alternate test, we supplemented glucose starvation conditions with galactose to promote oxidative phosphorylation.^55^ However, addition of neither MP nor galactose supplementation affected the cell viability rescue provided from glucose deprivation (Figure 5b).

In order to survive during the 24-h glucose starvation CQ treatment period, cells must utilise other nutrient sources. A fundamental feature of cancer cells is heavy reliance on glutamine.^24, 56, 57^ We hypothesised that 4T1 cells metabolise glutamine to survive the glucose starvation period. Again, in the controls, cells starved of glucose displayed increased survival in response to CQ and serum starvation (Figure 5c). However, further withdrawal of glutamine (in glucose- and serum-free conditions, with CQ) led to complete loss of cell viability. Glutamine-free starvation was notably not cytotoxic in the absence of CQ. These data highlight that 4T1 cells have a strong reliance on glutamine for survival, particularly when starved of other carbon sources such as glucose.

### CQ leads to lysosomal swelling via a glucose-dependent pathway

**Figure 6:**
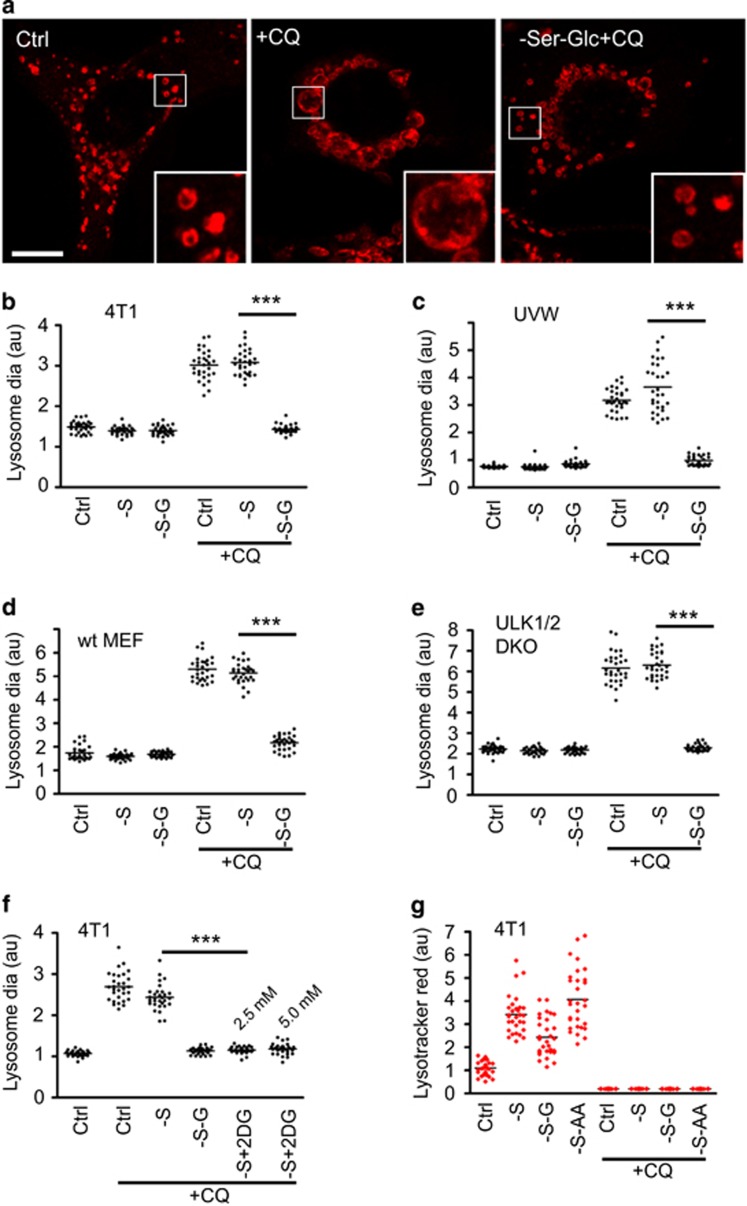
CQ induces lysosomal swelling, which is blocked upon glucose starvation. (**a**) 4T1 cells were treated with full-nutrient media (Ctrl); serum starvation; or serum and glucose starvation±CQ (25 *μ*M) as indicated for 8 h. Cells were fixed and stained for lysosomal-associated membrane protein-1 (LAMP-1). CQ induces robust lysosome swelling, but this is reversed upon glucose starvation, as highlighted in boxed insets. Scale bar: 10 *μ*m. (**b**) Quantification of lysosomal size in 4T1 cells described in (**a**). Average lysosomal diameters were measured for 30 cells per condition (each cell as a datum point, shown as relative arbitrary units). Reversal of CQ-induced lysosomal swelling by glucose starvation was conserved in: (**c**) UVW glioma cells, (**d**) wild-type MEF and (**e**) ULK1/2 DKO MEF. (**f**) 4T1 cells were treated with serum (and glucose starvation) or alternatively with 2DG (2.5 or 5 mM) ±CQ (25 *μ*M) as indicated for 8 h. Cells were quantified for lysosome swelling. (**g**) 4T1 cells were treated with full-nutrient media; serum starvation; or serum and glucose starvation ±CQ (25 *μ*M) as indicated for 4 h. Lysotracker red DND-99 (50 nM) was added for the final 30 min of treatment. Cells were analysed by confocal microscopy for lysotracker intensity (30 cells per condition). CQ deacidified lysosomes under all nutrient conditions. All data shown are representative of at least three independent experiments. ***P<0.001 by one-way ANOVA

As glucose starvation blocked CQ-mediated cell killing, we questioned whether effects of CQ on the lysosome were similarly glucose-dependent. We studied lysosomal morphology in cells following CQ treatment and nutrient starvation (Figure 6a). Control 4T1 cells showed abundant lysosomes with normal morphology. By contrast, CQ treatment produced markedly swollen lysosomes with strong LAMP-1 staining (Figure 6b). Serum starvation did not affect lysosomal enlargement caused by CQ. However, combined glucose and serum starvation fully blocked the ability of CQ to cause lysosomal swelling. We confirmed that solely glucose starvation was sufficient to reverse CQ-mediated lysosomal swelling (Supplementary Figure 3).

We examined whether the mechanism linking CQ efficacy and glucose metabolism was conserved. We tested UVW glioma cells, as CQ treatment has been used in glioblastoma therapy.^58, 59^ UVW cells exhibited similar lysosomal swelling following CQ treatment, which was further blocked by glucose starvation (Figure 6c). This inter-relationship was not limited to cancer cells. Wild-type and ULK1/2 DKO MEF showed typical normal-sized lysosomes under control and nutrient starvation conditions (Figures 6d and e). Here, CQ treatment also led to lysosomal swelling, which could be blocked by glucose starvation. Above, we showed that the glycolysis inhibitor, 2DG, rescued cytotoxicity driven by CQ. Therefore, we examined whether 2DG prevented CQ-mediated lysosomal swelling (Figure 6f). Indeed, 2DG, either at 2.5 and 5 mM, significantly blocked the ability of CQ to induce lysosomal swelling. Altogether, the data highlight how CQ induces a marked lysosomal enlargement in cancer cells, wild-type MEF and autophagy-deficient MEF. In each of these cell systems, glucose starvation blocks the ability of CQ to induce lysosomal swelling. Furthermore, 2DG is able to mimic glucose starvation to block CQ effects on the lysosome.

As glucose starvation prevented CQ from swelling the lysosome, we hypothesised that CQ might be unable to access the lysosomal lumen. For example, glucose starvation might deplete cellular energy and reduce the lysosomal acidification gradient required to trap CQ. To monitor lysosomal pH, we used Lysotracker Red to selectively stain acidic vesicles (Figure 6g). In 4T1 cells, serum starvation increased lysosomal acidification. Lysosomal acidification was still maintained upon further glucose or amino-acid starvation. Importantly, addition of CQ completely ablated Lysotracker Red staining under basal and all starvation conditions. Therefore, CQ was still effectively targeting lysosomal acidification, even under the glucose starvation condition that blocks lysosomal swelling.

### Doxorubicin cytotoxicity is enhanced by CQ but is independent of glycolysis

**Figure 7:**
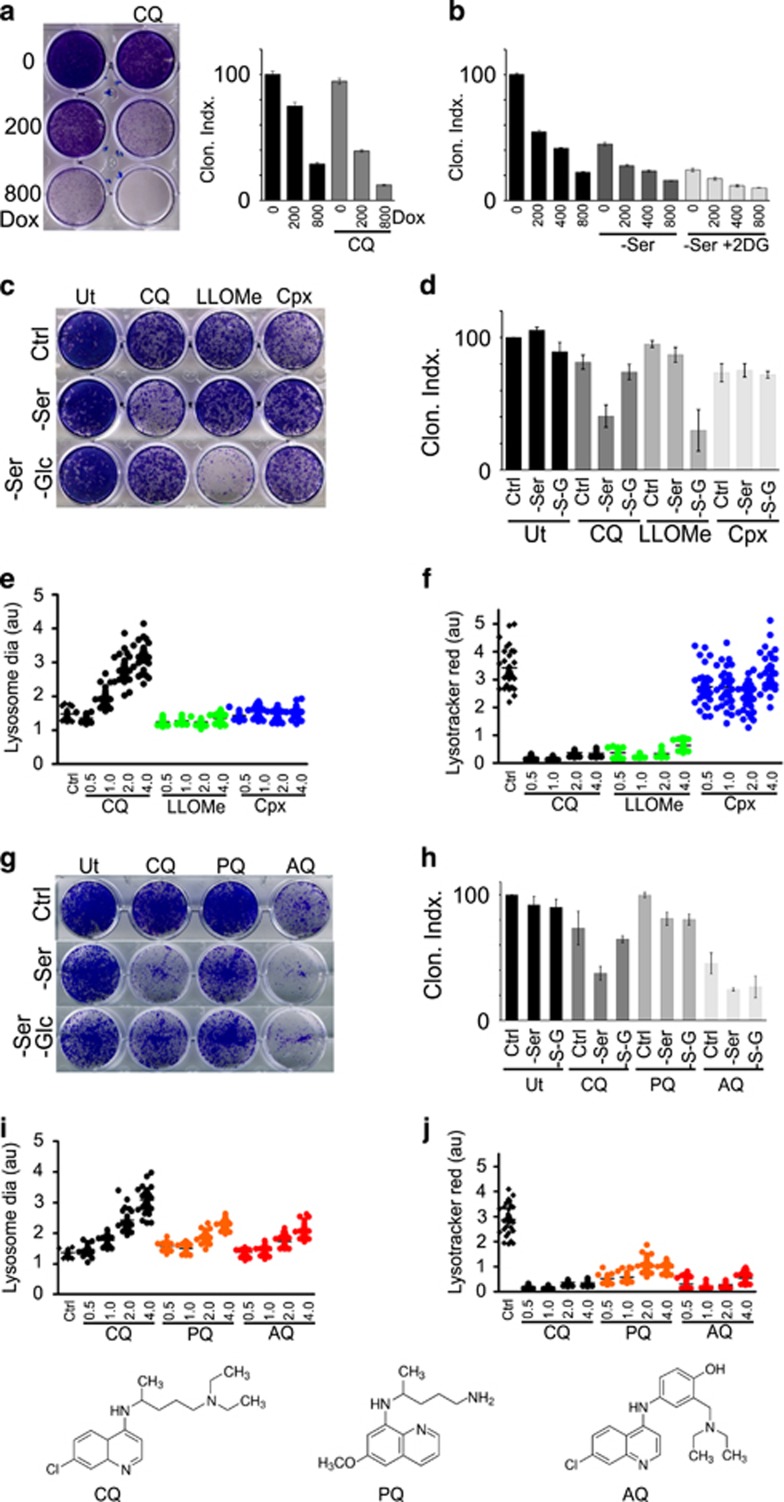
CQ cytotoxicity and lysosomal targeting are distinct from doxorubicin and alternate lysosomal membrane permeabilisation and quinoline compounds. (**a**) 4T1 cells were treated in full-nutrient media to doxorubicin (200/800 nM) in combination with CQ (25 *μ*M) as indicated for 24 h, followed by the clonogenic survival assay (*n*=4 ±S.E.M.). ***P<0.001; **P<0.01 by paired *t*-test. CQ sensitises to doxorubicin. (**b**) 4T1 cells were treated to doxorubicin in combination with serum starvation and 2DG (5 mM) as indicated for 24 h, followed by the clonogenic survival assay (*n*=4 ±S.E.M.). 2DG does not provide resistance to doxorubicin. (**c** and **d**) 4T1 cells were treated to full-nutrient media, serum starvation or serum and glucose starvation, in combination with: CQ (25 *μ*M), LLOMe (5 mM) or Cpx (150 *μ*g/ml), for 24 h, followed by assay for survival (*n*=3 experiments ±S.E.M.). (**e**) 4T1 cells were incubated in full-nutrient media (Ctrl) alone or in presence CQ, LLOMe or Cpx for the indicated times (hours) followed by analysis of LAMP-1 (lysosomal swelling; 30 cells per condition). All imaging data representative of at least three independent experiments. (**f**) Cells treated in parallel were analysed for lysotracker red staining. (**g** and **h**) 4T1 cells were treated with full-nutrient media; serum starvation; or serum and glucose starvation ±CQ, PQ or AQ (all 25 *μ*M) as indicated, for 24 h, followed by clonogenic cell viability measurements (*n*=3 experiments ±S.E.M.). (**i**) 4T1 cells were analysed for LAMP-1 lysosomal swelling as in (**c**; 30 cells per condition). CQ led to the most robust lysosomal swelling. (**j**) Cells treated in parallel were analysed for lysotracker red staining. CQ, AQ and PQ all rapidly led to lysosome deacidification

Doxorubicin is a widely used therapeutic in breast cancer, but lysosomal sequestration of this drug can lead to resistance.^60, 61^ As we observed that CQ disrupted severely lysosomal homeostasis, we investigated the interaction between these two forms of chemotherapy. CQ markedly enhanced the sensitivity of 4T1 cells to doxorubicin (Figure 7a). However, inhibition of the glucose flux with 2DG did not lead to any resistance towards doxorubicin (Figure 7b).

### Mechanism of CQ is distinct from classical lysosomal-membrane permeabilisation

The correlation between lysosomal swelling and cell death suggested lysosomal membrane permeabilisation (LMP), in line with previous data.^39^ LMP cell death is driven by leakage of cathepsins from the lysosome, thereby activating caspases or cathepsin-mediated breakdown of cellular components.^62, 63^ To examine lysosomal mechanisms, we compared CQ to two other compounds characterised for LMP. L-Leucyl-L-Leucine methyl ester (LLOMe) becomes converted into a bioactive form inside the lysosome to cause organellar disruption.^64, 65, 66^ The quinolone antibiotic ciprofloxacin (Cpx) has been shown to induce LMP and mitochondrion-dependent cell death.^67, 68^ The control samples here confirmed cytotoxicity from CQ+serum starvation, which was blocked by glucose starvation (Figures 7c and d). In contrast, LLOMe cell killing was not potentiated by serum starvation but was markedly enhanced only in the context of serum and glucose starvation. Cpx showed only mild effects on viability in 4T1 cells that was independent of nutrient starvation conditions.

As these three lysosomal targeting agents displayed different cytotoxic and nutrient-dependent profiles, we next interrogated their effects on the lysosome. CQ treatment induced lysosomal swelling in a time-dependent manner, progressing in severity over 4 h (Figure 7e). In contrast, neither LLOMe nor Cpx led to any lysosomal swelling. We further inspected lysosomal acidification. Both CQ and LLOMe treatments led to rapid (within 30 min) and complete deacidification of lysosomes (Figure 7f). In contrast, Cpx did not have clear effects on Lysotracker staining. In summary, our data indicate widely distinct profiles of lysosomal targeting by these lysosomotropic compounds. Only CQ led to time-dependent lysosomal swelling that was blocked by glucose starvation.

### Mechanism of AQ is distinct from CQ and is not glucose-dependent

CQ belongs to a family of antimalarial quinoline compounds, and related members are being widely explored as cancer therapeutics. AQ, a 4-aminoquinoline, and Primaquine (PQ), a 8-aminoquinoline, have been studied in melanoma and oral squamous carcinoma.^21, 69^ We questioned whether these structurally related compounds would also display an interaction with nutrient starvation (Figures 7g and h). When compared at equimolar levels, PQ was not cytotoxic alone but showed mildly increased potency when combined with serum starvation, although effects were not as strong as CQ. Furthermore, PQ action was not inhibited upon further starvation of glucose. By comparison, AQ was more potent than CQ when used alone and showed further cytotoxicity when combined with serum starvation. Glucose starvation did not show any protective rescue towards AQ. In terms of lysosomal targeting, both PQ and AQ led to similar time-dependent swelling of the lysosome but effects were not as robust as compared with CQ (Figure 7i). However, all three quinolines rapidly de-acidified the lysosome (Figure 7j). In summary, these three quinoline structural derivatives have markedly different effects on cell viability and lysosomal swelling. Furthermore, AQ was most potent in killing 4T1 cells and this cytotoxicity was not blocked by glucose starvation.

### CQ-mediated lysosomal damage and cell death are glycolysis-dependent

**Figure 8:**
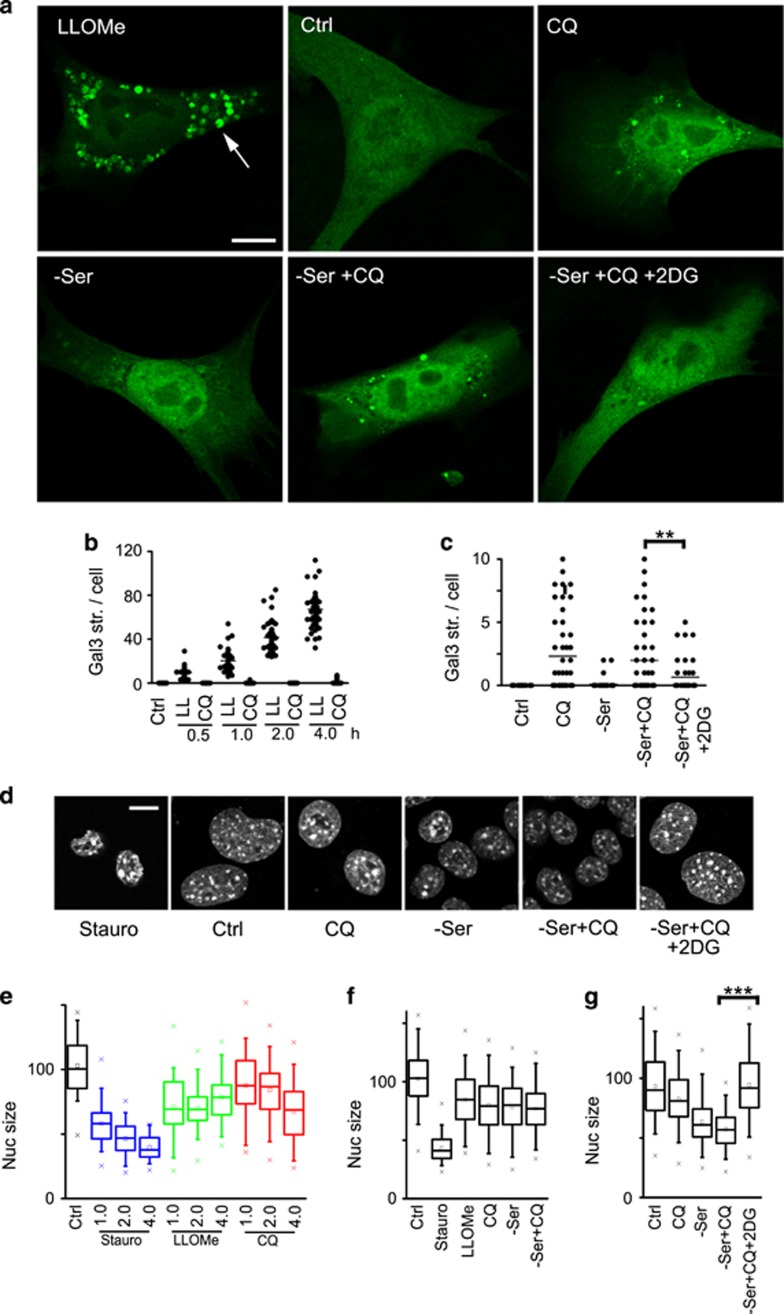
Inhibition of glycolysis rescues CQ-mediated lysosome damage and cell death. (**a**) 4T1 cells expressing GFP-Galectin3 were treated to CQ (25 *μ*M) in full-nutrient media or in combination with serum starvation (±2-deoxyglucose (5 mM)) as indicated for 18 h. As control, cells were treated with LLOMe (2.5 mM) for 2 h. Scale bar: 10 *μ*m. (**b**) GFP-Galectin3 puncta were quantified in 4T1 cells treated to LLOMe (2.5 mM) or CQ (25 *μ*M) for up to 4 h (50 cells from *N*=4 independent samples). (**c**) GFP-Galectin3 puncta were quantified in 4T1 cells treated as indicated for 18 h (50 cells from *N*=4 independent samples). **P<0.01 by paired *t*-test. 2DG inhibits lysosomal damage from CQ. (**d**) 4T1 cells were treated to CQ (25 *μ*M) in full-nutrient media or in combination with serum starvation (±2-deoxyglucose (5 mM)) as indicated for 18 h and nuclei were visualised. As control, cells were treated with staurosporin (1 *μ*M) for 2 h. Scale bar: 10 *μ*m. (**e**) Nucleus area was quantified in 4T1 cells treated as indicated for up to 4 h (45–80 cells per condition, representative of four experiments). (**f**) Nucleus area in 4T1 cells treated for 4 h (170–230 cells per condition from four experiments). (**g**) Nucleus area in 4T1 cells treated for 18 h (190–260 cells per condition from four experiments). ***P<0.001 by paired *t*-test. 2DG inhibits lysosomal damage from CQ

CQ-induced cytotoxicity was dependent on glucose metabolism and our results suggested lysosome-mediated cell death. To further dissect the mechanism, we investigated lysosome damage by studying galectin puncta formation. Galectin members bind to β-galactoside sugars such as those exposed upon LMP.^70^ Indeed, 4T1 cells treated with LLOMe formed extensive puncta of GFP-Galectin3 (Gal3) within 1–2 h of treatment (Figures 8a and b). Surprisingly, CQ treatment under the same timeframe did not generate comparably strong Gal3 puncta. However, prolonged CQ treatment led to Gal3-detectable lysosomal damage, both alone or with serum starvation (Figure 8c). Blocking glucose flux with 2DG significantly reduced CQ-dependent lysosome permeabilisation, both in 4T1 cells and MEF (Supplementary Figure 4).

In parallel, we monitored nuclear morphology, as this readout correlates with apoptosis and other forms of cell death. Staurosporin, as a positive control for apoptosis, led to strong nuclear condensation within 1–2 h of treatment (Figures 8d and e). In contrast, targeting the lysosome using LLOMe or CQ produced relatively weaker nuclear condensation under the same timeframe. CQ treatment combined with serum starvation led to higher levels of nuclear condensation, but only after prolonged treatment (Figures 8f and g). Interestingly, inhibition of glycolysis with 2DG completely reversed this cell death phenotype caused by CQ and serum starvation.

## Discussion

CQ is widely used in clinical trials, but its mechanism is still unclear. Although initially studied for DNA binding,^71, 72^ CQ is better recognised for its properties as a weak base, leading to uptake and deacidification of the lysosome.^25^ Block of lysosomal function thereby stops autophagy membrane trafficking and degradative flux. On the basis of the role of autophagy for cell metabolism and survival, the therapeutic efficacy of CQ in cancer has been widely proposed to be through autophagy inhibition.

Here, we show that CQ potently sensitises cancer cells to a number of different stressors, in agreement with the current clinical applications.^2^ In contrast, genetic inhibition of autophagy was not able to mimic CQ and drive cell killing. Autophagy is integral for intracellular quality control and metabolism.^1, 5^ However, our findings here indicate that blocking autophagy does not necessarily halt cancer cell growth. Furthermore, CQ-mediated cell killing was not modulated when autophagy was blocked, consistent with other data.^26, 28^

Surprisingly, in exploring nutrient stress, we found that glucose starvation blocked CQ-mediated cytotoxicity. Inhibition of glycolysis with 2DG also rescued cells from CQ. In our working model, CQ enters cells and deacidifies lysosomes within an hour of treatment. Subsequently, CQ leads to swelling of lysosomal compartments, which culminated over time into lysosomal damage and leakiness, thereby triggering cell death. Glucose starvation (or 2DG) completely prevented lysosomal swelling, damage and cell death activation. Amino-acid starvation did not produce a similar rescue effect. Glucose levels are critical for assembly and function of the lysosomal vacuolar-ATPase.^73^ However, lysosomes were clearly acidified even following glucose starvation, and further addition of CQ abolished this signal. Therefore, CQ was still able to target lysosome pH under glucose starvation, but was unable to drive further swelling and lysosomal damage. Nonetheless, effects of CQ in patients may be limited inside the cores of tumours encountering limited vascularisation and glucose-deprivation metabolic stress.^74, 75, 76^ Thus, glucose limitation as a cause of resistance can apply to CQ, as seen with other chemotherapeutic compounds.^77, 78^

Intriguingly, marked lysosomal swelling was unique to CQ. The compound LLOMe, which has been widely used to trigger LMP,^64, 66^ led to more rapid lysosomal damage without swelling. Furthermore, LLOMe cytotoxicity was not blocked, but rather, enhanced by glucose starvation. Therefore, CQ promoted a slower form of LMP distinct from those described previously.^64, 67^ To identify improved anticancer strategies, another approach has been to re-purpose CQ-related antimalarial quinolines.^8, 21, 22, 79^ Here, we found a clear structure activity relationship when studying AQ, which resembles CQ, but with addition of a phenolic group on the tertiary amine side chain. As AQ had higher cytotoxicity and a lack of interference by glucose starvation, it may be a more robust compound with efficacy across the wider spectrum of cancer metabolic contexts. Interestingly, CQ, AQ and PQ all seem to target lysosome acidification similarly, but additional chemical moieties prevent AQ and PQ from producing a marked lysosomal swelling. We thus highlight how cytotoxicity is produced via mechanisms that differ between CQ and AQ.

## Materials and methods

### Cell culture

4T1 cells were purchased from the American Type Culture Collection (ATCC distributed via LGC Standards, Middlesex, UK; ATCC CRL-2539) and cultured in Dulbecco’s modified eagle medium (DMEM) with 4.5 g/l glucose (Lonza, Slough, UK, #BE12-614F) supplemented with 10% fetal bovine serum (Labtech International Ltd, East Sussex, UK, #FCS-SA), 4 mM L-Glutamine (Lonza #BE17-605E) and 100 U/ml penicillin/streptomycin (Lonza #DE17-602E) in a humidified incubator with 5% CO_2_ at 37 °C. Both MEFs and UVW glioma cells^80^ were cultured using the same method. Wild-type and ULK1/2 DKO MEF have been described.^38^ ATG7 mouse pLKO shRNA (TRCN0000092163) and pLKO scrambled shRNA (Addgene (#1864)) were used to transduce 4T1 cells, followed by selection with puromycin.

### Clonogenic survival assays

Cells (5 × 10^3^/ml) were plated in six-well cell culture plates (or in some cases 12 well). 24 h after seeding, fresh full nutrient growth media was replenished. Following a further 24 h, cells were stimulated for 24 h with the following nutrient starvation conditions ±CQ (25 *μ*M; Sigma-Aldrich Ltd., Gillingham, UK, #C6628). For serum starvation, full nutrient DMEM was replaced with DMEM (containing 4.5 g/l glucose, 4 mM L-glutamine and 100 U/ml penicillin/streptomycin as above) with no FBS. For glucose starvation, we used glucose-free DMEM media containing 4 mM L-Glutamine (Gibco - Life Technologies Ltd, Paisley, UK, #11966-025) but lacking FBS and penicillin/streptomycin. For amino starvation, DMEM was replaced with Earle’s balanced salt solution (EBSS; Sigma #E2888). We assessed the role of glutamine using serum, glucose and glutamine-free DMEM (ThermoFisher Scientific- Life Technologies Ltd, Paisley, UK #A1443001). We also assessed MP (Sigma #371173) and galactose (Sigma #G0750) as alternative carbon sources. Inhibitors tested in cells were as follows: 2DG (Sigma #D8375); RIP kinase 1 inhibitor Necrostatin-1 (Sigma #N9037); doxorubicin (Tocris Bioscience, Bristol, UK, #2252) and caspase 3 inhibitor z-DEVD-FMK (Tocris #2166). We used freshly prepared stock solutions of *N*-acetyl cysteine (Sigma #A7250). We compared alternative quinoline compounds: PQ (Sigma #160393) and AQ (Sigma #A2799) made up in sterile water as vehicle. We compared effects against LLOMe (Sigma #L7393) and Cpx (Sigma #17850).

To examine ionising irradiation, following 2 h pretreatment with CQ (25 *μ*M) in normal full-nutrient DMEM, cells were exposed to 1, 4 or 10 Gy doses in an X-Rad 225 irradiator (RPS services, Surrey, UK). Twenty-four hours post stress, media were replenished. We tested NVP-BEZ-235 at a final concentration range of 50–200 nM, ±CQ, diluted in normal full-nutrient DMEM. After 24 h stress conditions ±CQ, cells were changed back into full-nutrient DMEM and left to grow for 3 days. At end point, cells were stained with Giemsa followed by quantitation by solubilising stained cells in 30% acetic acid and measurement of absorbance (560 nm), essentially as described.^29^

### Western blot analysis

Cell lysates were analysed as described previously^38^ using the following antibodies: LC3: Mouse monoclonal (clone 5F10; Nanotools antibodies GmbH, Teningen, Germany, #0231-100); p62/SQSTM1: Guinea pig polyclonal (Progen Biotechnik GmbH, Heidelberg, Germany, #GP62-C); ATG7: Rabbit monoclonal (D12B11; Cell Signaling Technologies, New England Biolabs, Hitchin, UK, 8558); Caspase 3: Rabbit monoclonal (8G10; Cell Signalling #9665); and PARP: Mouse monoclonal (BD Biosciences, Oxford, UK, #556362).

### Microscopy

Cells were plated on glass coverslips (precoated with fibronectin (Sigma #F1141) in the case of 4T1 cells). After treatments, cells were fixed and stained using the following antibodies: LC3: Rabbit monoclonal (Cell Signalling 2775); p62/SQSTM1 (as above); or LAMP-1 (CD107a): Rat monoclonal (BD Biosciences #553792). Cell images were captured by confocal microscopy (Leica, SP5 (Leica Microsystems Ltd, Milton Keynes, UK); or alternatively by epifluorescence microscopy; Nikon Eclipse E600 (Nikon UK Limited, Surrey, UK). To quantify lysosomal size, diameters were measured for 10 lysosomes per cell, 30 cells per condition. Final data shown are representative of at least three independent experiments.

To detect lysosomal acidification, 4T1 cells were treated as indicated with 50 nM Lysotracker Red DND-99 (LTR; Thermo Fisher - Life Technologies Ltd, Paisley, UK, #L7528) added during the final 30 min of incubation. Cells were fixed with paraformaldehyde and immediately imaged using confocal microscopy. LTR intensity was quantified within cytoplasmic region of interests. Thirty cells per condition were analysed and data shown are representative of at least three independent experiments. To detect lysosomal membrane damage, 4T1 cells or MEF were transfected with pEGFP-hGal3 (gift from Tamotsu Yoshimori; Addgene (Cambridge, MA, USA) plasmid #73080^66^) using Lipofectamine 2000 and studied 24 h later. After treatments, cells were fixed followed by confocal imaging and quantification of puncta. To detect condensation of nuclei, cells were stained with Hoescht 33342 after treatments. Nucleus sizes were quantified from confocal images using Thresholding and the Analyze Particle function in ImageJ.

For live cell tracking, 4T1 cells were plated in 24-well dishes, treated as indicated and analysed using Incucyte Zoom (Essen Bioscience, Ltd., Welwyn Garden City, UK) at standard 5%CO_2_ at 37 °C, with quantification using Incucyte Cell Confluence measurements. We measured cell growth over the 24-h starvation stress period followed by 24 h after replenishment with full-nutrient drug-free media. Data points are average of two wells from a representative of two experiments.

### Statistical analysis

Quantitative data were managed in GraphPad Prism (GraphPad Software, Inc., La Jolla, CA, USA). We used one-way ANOVA with Tukey post test or Student’s paired *t*-test as appropriate.
