# Supplementary material for: Lysosomotropism depends on glucose: a chloroquine resistance mechanism
Source: Cell Death Dis. 2017 Aug 24;8(8):e3014–. doi: 10.1038/cddis.2017.416 (PMC5596595; doi:10.1038/cddis.2017.416)
Supplement: Supplementary Figure 1 [file cddis2017416x1.pdf]

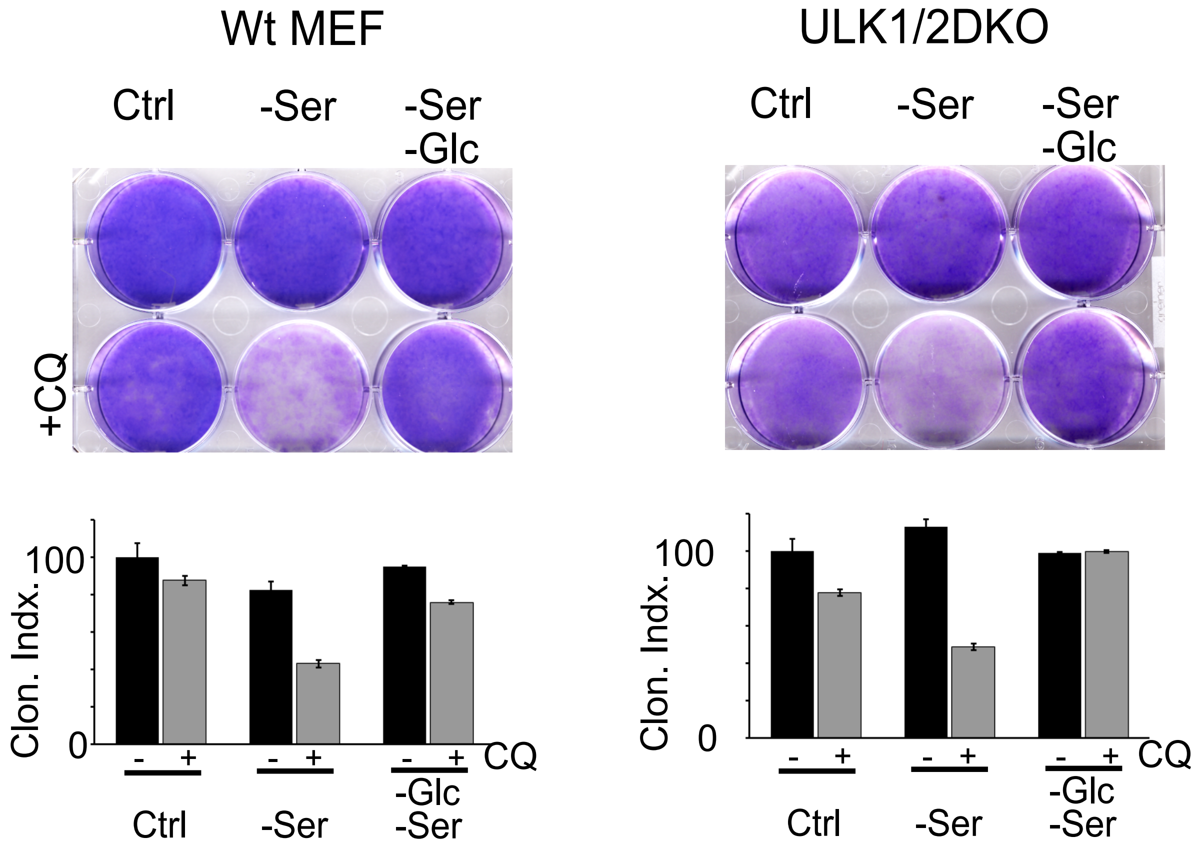

Supplemental Figure 1. Glucose starvation suppresses chloroquine-mediated cytotoxicity in MEF.

(A) Wildtype or ULK1/2 DKO MEF cells were exposed to serum starvation (-Ser) or combined glucose and serum starvation (-Ser-Glc) +/- CQ (25µM) as indicated for 24hrs. After this treatment, drug-free media was replenished and viability was assessed by clonogenic growth and quantified. Serum starvation combined with CQ killed MEF but this was blocked by glucose starvation. Cell death from CQ and rescue upon glucose starvation did not depend on ULK1/2 mediated autophagy.
