# Supplementary material for: Lysosomotropism depends on glucose: a chloroquine resistance mechanism
Source: Cell Death Dis. 2017 Aug 24;8(8):e3014–. doi: 10.1038/cddis.2017.416 (PMC5596595; doi:10.1038/cddis.2017.416)
Supplement: Supplementary Figure 2 [file cddis2017416x2.pdf]

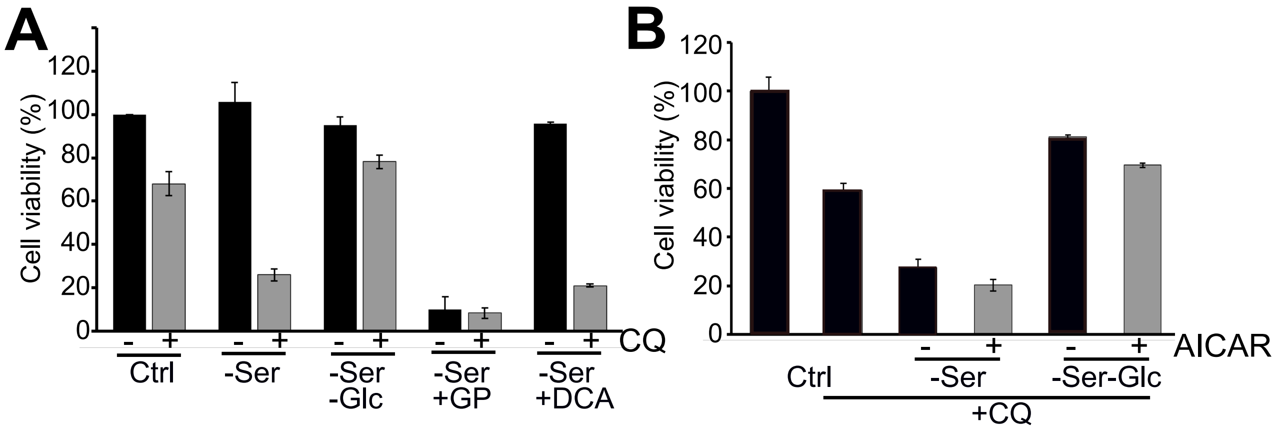

Supplemental Figure 2. Rescue of cell viability is not produced by general glycolysis inhibitors and is independent of AMPK.

(A) We tested if other inhibitors of glycolysis conferred the same protective advantage as 2DG. The anti-cancer natural compound Gossypol can inhibit glycolysis via inhibition of lactate dehydrogenase, thereby causing a build-up of pyruvate. Dichloroacetate (DCA) inhibits pyruvate dehydrogenase kinase, which normally phosphorylates and inactivates pyruvate dehydrogenase (PDH). DCA promotes higher PDH activity and conversion of pyruvate to acetyl-CoA, shifting energy generation towards oxidative phosphorylation and away from glycolysis. Cells were treated with serum-free DMEM + Gossypol (20μM) or dichloroacetate (5mM) +/- CQ (25μM) as indicated, for 24 hrs, followed by cell viability measurements. Addition of Gossypol could not prevent CQ combined with serum starvation from killing cells. Moreover, Gossypol was cytotoxic in serum starved cells, even without CQ treatment. Gossypol has been shown to act as a BH3 mimetic to initiate autophagy-associated cell death, and so this small molecule is pleiotropic (Lian et al, 2010; Yuan et al, 2013). In 4T1 cells, the overall cytotoxic effects of Gossypol were predominant and so no rescue of cell viability could be detected. DCA was not able to rescue cell death caused by CQ and serum starvation. Therefore, inhibition of glycolysis at an early stage using 2DG has been the best pharmacologic agent that mimics glucose starvation in preventing cell killing by CQ.

(B) Glucose starvation and energy depletion activates an AMPK-dependent metabolic checkpoint leading to cell-cycle arrest, but this response also promotes cell survival. We tested whether increased cell survival upon glucose starvation was driven by AMPK. Cells were treated with serum-free DMEM or serum- and glucose-free DMEM + CQ (25μM) for 24hrs, where incubated in the presence of AICAR (1mM), followed by cell viability measurements. Co-incubation with AICAR (activating AMPK) could not rescue cell viability following CQ-treatment and serum starvation. AICAR also did not provide any further improvement on cell viability above that offered by glucose starvation.

Lian, J., Wu, X., He, F., Karnak, D., Tang, W., Meng, Y., Xiang, D., Ji, M., Lawrence, T. S. and Xu, L. (2010). A natural BH3 mimetic induces autophagy in apoptosis-resistant prostate cancer via modulating Bcl-2–Beclin1 interaction at endoplasmic reticulum. Cell Death and Differentiation, 18: 60-71. doi:10.1038/cdd.2010.74.

Yuan, Y., Tang, A. J., Castoreno, A. B., Kuo, S. Y., Wang, Q., Kuballa, P., Xavier, R., Shamji, A. F., Schreiber, S. L. and Wagner, B. K. (2013). Gossypol and an HMT G9a inhibitor act in synergy to induce cell death in pancreatic cancer cells. Cell Death Dis, 4: e690. doi:10.1038/cddis.2013.191.
