# Supplementary material for: Lysosomotropism depends on glucose: a chloroquine resistance mechanism
Source: Cell Death Dis. 2017 Aug 24;8(8):e3014–. doi: 10.1038/cddis.2017.416 (PMC5596595; doi:10.1038/cddis.2017.416)
Supplement: Supplementary Figure 3 [file cddis2017416x3.pdf]

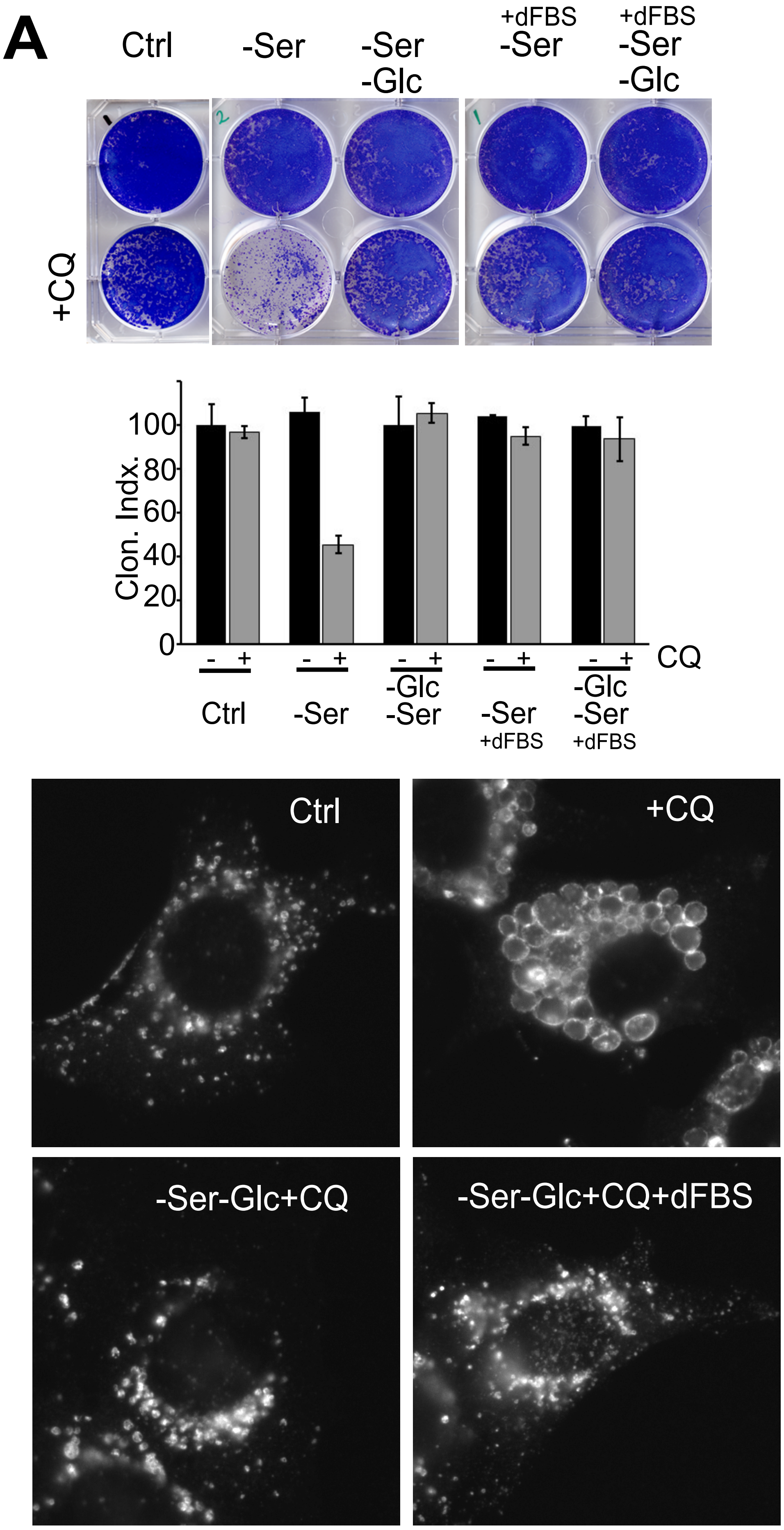

Supplemental Figure 3. Glucose starvation alone suppresses lysosomal swelling caused by chloroquine.

(A) 4T1 cells were exposed to serum starvation (-Ser) or combined glucose and serum starvation (-Ser-Glc) +/- CQ (25μM) as indicated for 24hrs. To test role of glucose, 10% dialysed FBS was supplemented as indicated. After this treatment, drug-free media was replenished and viability was assessed by clonogenic growth and quantified. Serum starvation combined with CQ killed cells but this was blocked by glucose starvation. As expected in this control experiment, addition of dialysed FBS provided survival growth factors and rescued cell death
(B) 4T1 cells were exposed to the indicated conditions for 8 hrs and assayed for lysosomal morphology by staining for LAMP-1. CQ leads to lysosomal swelling, which is reversed by concurrent glucose (and serum) starvation. Addition of 10% dialysed FBS to this condition had no effect and lysosomal swelling was still inhibited. Therefore, starvation of glucose alone prevented lysosomal swelling caused by CQ.
