# Supplementary material for: Lysosomotropism depends on glucose: a chloroquine resistance mechanism
Source: Cell Death Dis. 2017 Aug 24;8(8):e3014–. doi: 10.1038/cddis.2017.416 (PMC5596595; doi:10.1038/cddis.2017.416)
Supplement: Supplementary Figure 4 [file cddis2017416x4.pdf]

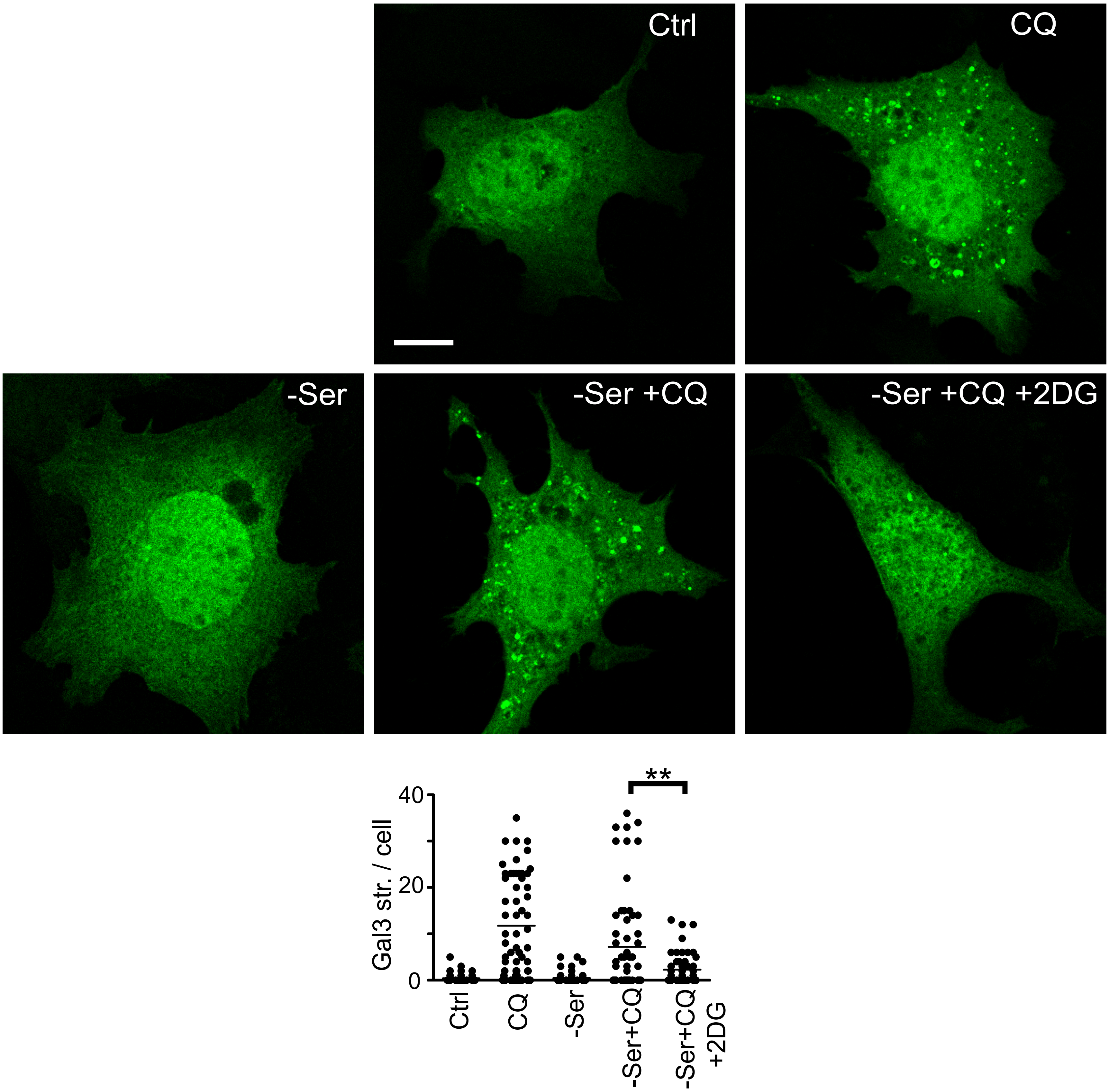

Supplemental Figure 4. Inhibition of glycolysis rescues CQ-mediated lysosome damage.

Wildtype MEF expressing GFP-Galectin3 were treated to CQ (25μM) in full-nutrient media or in combination with serum starvation (+/-2-deoxyglucose (5mM)) as indicated for 18hrs. Scale bar: 10 μm. GFP-Galectin3 puncta were quantified in MEF as indicated for 18hrs (60 cells from N=3 samples). (**) $P < 0.01$ by paired t-test. 2DG inhibits lysosomal damage from CQ.
