# Supplementary material for: Lysosomotropism depends on glucose: a chloroquine resistance mechanism
Source: Cell Death Dis. 2017 Aug 24;8(8):e3014–. doi: 10.1038/cddis.2017.416 (PMC5596595; doi:10.1038/cddis.2017.416)
Supplement: Supplementary Movie Legend [file cddis2017416x5.pdf]

Gallagher et al Supplemental movie data

We monitored 4T1 cell morphology by hourly live-cell imaging during nutrient and CQ treatments (see corresponding still images and quantification in Figure 4).

1. Untreated control
2. CQ (25μM)
3. CQ (25μM) in serum-free DMEM
4. CQ (25μM) in serum- and glucose-free DMEM

After 24 hrs of drug and stress treatments, full-nutrient drug-free media was replenished. Full field-of-view movie are shown for the entire 48 hr experiment. Zoomed movies also shown for the first 23 hrs of stress treatment.
